# Endoscopic and Histopathological Characteristics of Gastrointestinal Lymphoma: A Multicentric Study

**DOI:** 10.3390/diagnostics13172767

**Published:** 2023-08-26

**Authors:** Quang Trung Tran, Thinh Nguyen Duy, Bao Song Nguyen-Tran, Tung Nguyen-Thanh, Quy Tran Ngo, Nam Phuong Tran Thi, Vi Le, Thuan Dang-Cong

**Affiliations:** 1 Department of Internal Medicine A, University Medicine Greifswald, 17475 Greifswald, Germany; quangtrung.tran@med.uni-greifswald.de; 2 Gastroenterology-Endoscopy Center, Hue University of Medicine and Pharmacy, Hue University, Hue 49000, Vietnam; 3 Faculty of Medicine and Pharmacy, Tay Nguyen University, 567 Le Duan Street, Buon Ma Thuot 63000, Vietnam; ndthinh@ttn.edu.vn; 4 Department of Histology, Embryology, Pathology and Forensic, University of Medicine and Pharmacy, Hue University, 6 Ngo Quyen Street, Hue 49000, Vietnam; ntbsong@huemed-univ.edu.vn (B.S.N.-T.); nqtran@huemed-univ.edu.vn (Q.T.N.); ttnphuong@huemed-univ.edu.vn (N.P.T.T.); levi96.res@gmail.com (V.L.); 5 Faculty of Basic Science, University of Medicine and Pharmacy, Hue University, 6 Ngo Quyen Street, Hue 49000, Vietnam; nttung@huemed-univ.edu.vn; 6 Department of Pathology, Khanh Hoa Oncology Hospital, 229 Nguyen Khuyen Street, Nha Trang 57000, Vietnam

**Keywords:** gastrointestinal lymphoma, endoscopic characteristics, histopathology

## Abstract

***Background***: Extranodal non-Hodgkin lymphoma (NHL) is more prevalent in the gastrointestinal (GI) tract than in other sites. This study aimed to determine the endoscopic characteristics of primary gastrointestinal non-Hodgkin lymphomas. ***Methods***: We investigated 140 patients from three tertiary referral hospitals with primary malignant lymphoma of the gastrointestinal tract. Characteristics of the lesions were evaluated and analyzed using image-enhanced endoscopy, endoscopic ultrasound, and histopathology. ***Results***: The median age was 60.5 (range: 11–99), and 59 (42.1%) were female. The most frequent complaint was abdominal pain (74.3%), followed by bloody feces (10%) and diarrhea (2.9%). B symptoms were observed in 15 (10.7%) patients. GI obstruction was the most common complication (10.0%), followed by hemorrhage (7.9%) and perforation (1.5%). Regarding endoscopic findings, the identified sites were the following: the stomach (61.4%), colon (10%), small intestine (10%), ileocecum (8.6%), rectum (6.4%), and duodenum (3.6%). Diffuse large B-cell lymphoma (DLBCL) and mucosa-associated lymphoid tissue (MALT) lymphoma are most prevalent in the stomach. *Helicobacter pylori* was identified in 46 cases (39.0%), with MALT lymphoma being the most infected subtype. Nearly all gastrointestinal non-Hodgkin lymphomas manifested as superficial type (25–59.6%) and ulcer type (15.6–50%) under endoscopy. We found that fungating type and protruding with ulcer type were more frequent types of aggressive lymphomas (diffuse large B-cell lymphoma, mantle cell lymphoma, and T-cell lymphoma) compared to the indolent types (MALT lymphoma, follicular lymphoma, duodenal-type follicular lymphoma, and small lymphocytic lymphoma) (*p* < 0.05). ***Conclusions***: This study showed that most subtypes of gastrointestinal non-Hodgkin lymphomas exhibited same endoscopic features (superficial type and ulcer type). Aggressive gastrointestinal non-Hodgkin lymphomas (diffuse large B-cell lymphoma, mantle cell lymphoma, and T-cell lymphoma) were highly suspected when fungating lesions and protruding with ulcer lesions were encountered under endoscopy. Endoscopists should be aware of the connection between enhanced endoscopic characteristics and histological varieties of gastrointestinal lymphoma to improve diagnosis.

## 1. Introduction

Primary gastrointestinal (GI) lymphomas are malignancies that originate from the lymphocytes of the GI tract. The digestive tract is the most frequent extranodal location of non-Hodgkin lymphomas. However, gastrointestinal lymphoma is a rare tumor, comprising only 10–15% of non-Hodgkin lymphomas and 1–4% of GI tumors [1,2,3]. Although this tumor can develop in any part of the digestive tract, the stomach is the most affected (in approximately 2/3 of the cases), followed by the small intestine and other areas. Diagnosis of gastrointestinal lymphoma is challenging. Endoscopic findings and histopathological analysis are indispensable for diagnosing and distinguishing malignant from benign lesions [1,4]. Specifically, the proportion of GI lymphomas initially detected by endoscopists is growing owing to the development of endoscopic technology. Owing to their nonspecific appearance during endoscopy, there is no universal classification standard at the macroscopic level. Various authors have proposed endoscopic classification schemes for GI lymphomas [3,5,6]. Due to the above non-specific endoscopic findings, histopathological examination is required for the diagnosis of GI lymphoma [3,7].

Since the publication of the 4th edition of the World Health Organization classification of lymphoid neoplasms, there have been significant advances and in-depth insights into molecular genetics and biological and clinical features of hematologic malignant neoplasms, necessitating an update in 2016 prior to the formal publication of the 5th edition as part of the WHO “blue book” series [8]. The recent classification includes several significant revisions, including modifications and updates to the classification of digestive tract lymphoma. Histologically, B-cell non-Hodgkin lymphomas comprise the vast majority of gastrointestinal (GI) lymphomas, with diffuse large B-cell lymphoma (DLBCL) and marginal zone lymphoma of mucosa-associated lymphoid (MALT lymphoma) being the most prevalent type, whereas T-cell lymphomas are not as common, accounting for only 4% to 6% [7,9]. In addition, it has been observed that the subtype and prevalence of a particular lineage varies based on the location of the digestive tract. In particular, MALT lymphoma and DLBCL tend to affect the stomach, whereas mantle cell lymphoma (MCL) is typically found in the terminal ileum, jejunum, and colon; follicular lymphoma and enteropathy-associated T-cell lymphoma are found in the small intestine [10,11]. 

In this research, we sought to determine the endoscopic characteristics of primary gastrointestinal non-Hodgkin lymphomas and the associations between endoscopic appearance and histopathological types of gastrointestinal lymphomas in a cohort of Vietnamese patients.

## 2. Patients and Methods

### 2.1. Patient Characteristics

We conducted descriptive, retrospective, multicentric research to investigate the endoscopic findings and histopathological features of primary gastrointestinal lymphomas diagnosed at the Hue Central Hospital, Hue University of Medicine and Pharmacy Hospital, and Vietnam National Cancer Hospital between January 2020 and June 2022. This study was approved by the Institutional Ethics Committee of Hue University of Medicine and Pharmacy (approval number: H2022/016). A definitive diagnosis of gastrointestinal lymphoma was made based on immunohistochemical analysis. Data on age, sex, chief complaint, B symptoms, lactate dehydrogenase (LDH) levels, complications, stage, treatment, lesion location, tumor size, endoscopic discovery, and histopathologic features were collected retrospectively from the databases of each hospital. Primary gastrointestinal lymphoma was identified using Dawson’s criteria, which include the following: (1) no peripheral lymphadenopathy at the time of detection, (2) no enlarged mediastinal lymph nodes, (3) normal total and differential white blood cell count, (4) predominance of bowel lesion at laparotomy with only nearby lymph nodes affected, and (5) no lymphomatous involvement of liver and spleen [12]. 

### 2.2. Endoscopic Examinations 

Patients with GI symptoms were examined using endoscopic procedures by endoscopists with >10 years of experience. These techniques include esophagogastroduodenoendoscopy, colorectal endoscopy, small-bowel enteroscopy, magnifying endoscopy with narrow-band imaging (NBI), and endoscopic ultrasound. The lesions were described using endoscopic terms according to Kanno and colleagues [3] including superficial, protruding without ulcer, fungating, protruding with ulcer, giant fold, and multiple nodule forms. Biopsy specimens were obtained from deep into the lamina propria, with a size of at least 3 mm. The status of *H. pylori* infection was confirmed via the results of at least one of two tests: urease test using biopsy specimen or a 14C-urea breath test.

### 2.3. Histopathological Examination and Pathological Classification

Pathological specimens were either endoscopic biopsies or surgical resections. Based on the histological morphology and immunohistochemistry results, pathological examination was performed. The immunohistochemical marker combinations chosen were CD20, CD79a, Bcl6, Bcl2, CD10, CD3, CD5, Cyclin D1, CD23, Ki67, Mum1, and AE13, based on morphological orientation. Regardless of whether T-cell lymphoma is suspected, CD4, CD8, and CD56 levels can also be observed. Other markers were selected based on these circumstances. Experienced pathologists classified the diagnoses and phenotypes according to the fifth edition of the WHO classification of digestive system tumors [13].

### 2.4. Statistical Analysis

Google Sheets and RStudio 2022.12.0/R 4.1.2. [14] was used for statistical analysis. Descriptive statistics were used for categorical and continuous variables. The correlation between *H. pylori* infection and lesion location/endoscopic findings was determined using the Fisher–Freeman–Halton test. The level of significance was established at 5%.

## 3. Results

### 3.1. Patient Characteristics

A total of 140 patients with primary gastrointestinal lymphoma were diagnosed and phenotyped using IHC, and 59 (42.1%) were female. The median age was 60.5 (11–99). The chief complaints were abdominal pain in 104 patients (74.3%), bloody stools in 14 (10.0%), diarrhea in 4 (2.9%), and other complaints in 18 (12.9%). B symptoms (also known as systemic symptoms, are a group of symptoms associated with non-Hodgkin lymphoma and indicate a more advanced stage of the disease) were observed in 15 (10.7%) patients. The mean LDH was 255.6 U/L (range: 91–2199). Complications included hemorrhage in 11 patients (7.9%), obstruction in 14 (10.0%), and perforation in 2 (1.4%). The disease stage was I in 72 patients (51.4%), II in 26 patients (18.6%), III in 10 patients (7.1%), and IV in 32 patients (22.9%). Treatment therapies were surgical alone in six patients (4.3%), non-surgical in 52 patients (37.1%), both in 46 patients (32.9%), and supportive in 36 patients (25.7%).

### 3.2. Endoscopic Findings

Gastrointestinal lymphomas are most frequently found in the stomach, followed by the colon, small intestine, ileocecum, rectum, and duodenum. The largest size of the lesion was found in the rectum (46.3 ± 26.7 mm), and its smallest counterpart was in the duodenum (24.0 ± 6.5 mm) (Table 1). 

**Figure 1:**
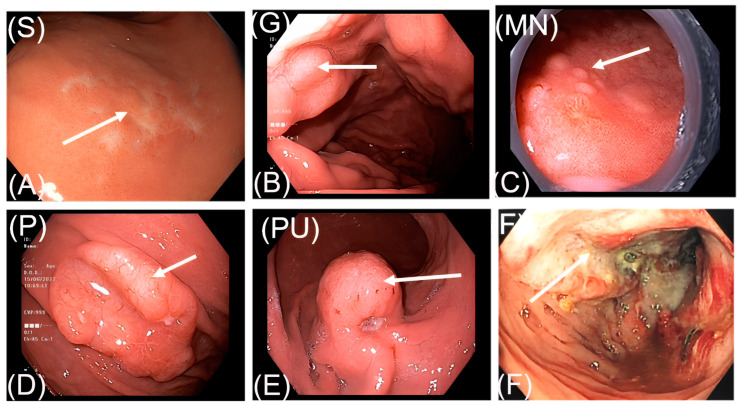
Endoscopic features of gastrointestinal lymphoma. (**A**). S: superficial form; (**B**): G: giant fold form; (**C**): MN: multiple nodule form; (**D**): P: protruding without ulcer form; (**E**): PU: protruding with ulcer form; (**F**): fungating form.

More than half of the gastrointestinal lymphoma lesions observed during endoscopy were superficial (51.4%). Protruding without lymphoma ulcer, fungating, and protruding with ulcer type were also common, accounting for 20.7%, 12.9%, and 9.3%, respectively. Giant folds and multiple nodules were the two rare features, accounting for 3.6% and 2.1%, respectively (Table 2). The endoscopic illustrations of these six subtypes among our patients can be seen in Figure 1. 

**Figure 2:**
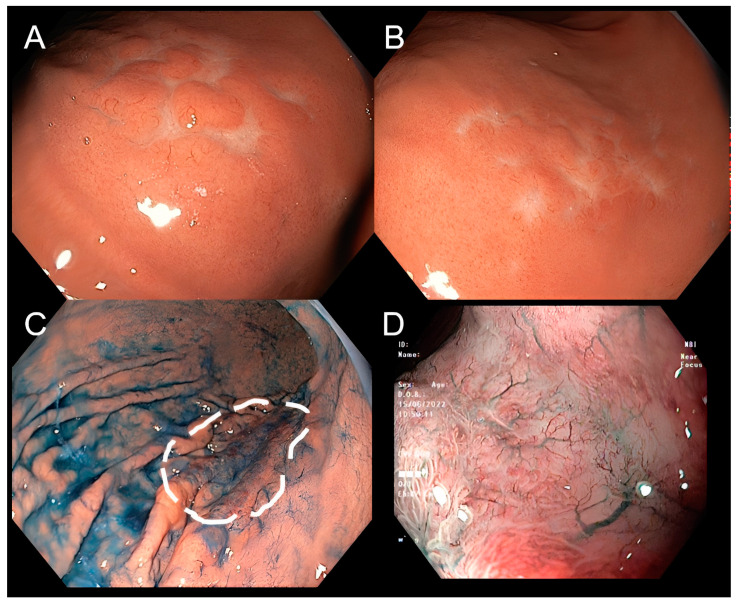
Lymphoma on narrow-band imaging (NBI) and magnifying endoscopy. Conventional endoscopy shows a superficial stomach lesion with tiny nodules resembling cobblestones (**A**,**B**). Using procedures including chromoendoscopy by indigo carmine can more clearly delineate the margin of the lesion (**C**). Using narrow-band imaging (NBI) and magnifying endoscopy, the lesion has no clear boundaries, and there is at surface-inverted microstructure along with disappeared gastric pits, and with tree-like vessels pattern (**D**).

Out of the 20 patients who underwent image-enhanced endoscopy technologies, a majority were diagnosed with MALT-lymphoma with tree-like or branch-like microvessel pattern (Figure 2). Among these magnified NBI cases, 13 patients have lesions in the stomach, while 7 cases were located in other parts of the gastrointestinal tract. Among the patients, 11 (55%) were diagnosed with MALT lymphoma, and the tree-like or branch-like microvessel pattern was observed in 72.7% of all cases. On the other hand, there were four cases of non-MALT lymphoma that exhibited the aforementioned “tree-like or branch-like microvessel pattern”, accounting for 20% of cases. 

### 3.3. Relationship between Endoscopy and H. pylori Infection of Gastrointestinal Lymphoma

*H. pylori* was found in 48 cases (38.7%). Patients with lymphomas in the stomach, ileocecum, or duodenum are more likely to be affected by *H. pylori* infection than those with other gastrointestinal tract lymphomas. However, differences in the proportion of *H. pylori* negative and positive were observed only in the colon and small intestine (*p* = 0.001 and 0.016, respectively). There was also a statistically significant correlation between *H. pylori* infection and lymphoma location in the digestive tract (*p* = 0.015) (Table 3).

MALT lymphoma, in total, did not show a statistically significant difference between the two groups of *H. pylori*. However, when divided into two groups of the stomach and other locations, *H. pylori* was revealed predominant in the stomach group at 68.2%, while its counterpart was only 20.0% (*p* = 0.021). *H. pylori* was not present in SLL, TCL, or D-FL. Overall, the *H. pylori* infection and pathological subtypes, however, had no statistically significant relationship in these data (Table 4).

Generally, the majority of pathological subtypes of gastrointestinal lymphoma lacked *H. pylori* infection, except for the MALT and FL subtypes, which had *H. pylori* infection in 53.1% (17 cases) and 100% (1 case), respectively. The proportion of *H. pylori* among DLBCL patients was statistically significant (*p* = 0.02).

### 3.4. Relationship between Endoscopy and Pathological Subtypes of Gastrointestinal Lymphoma

The most frequent location of DLBCL (59/82) and MALT lymphoma (22/32 cases) was the stomach. There were no cases of MALT lymphoma in the ileocecum or DLBCL in the duodenum. MCL was observed in almost all studied locations with approximately equal proportions. FL was found only in the stomach (one case) and ileocecum (one case). One case of SLL was present in the stomach and one case in the rectum. No TCL was found in the rectum or duodenum. The correlation between lesion location and pathological subtype was statistically significant (*p* < 0.001). There was also a significant difference in the number of patients with each subtype in the stomach (*p* < 0.001). Owing to the low expected frequencies at other locations, the *p*-value could not be calculated (Table 5).

Aggressive lymphomas include diffuse large B-cell lymphoma, mantle cell lymphoma, and T-cell lymphoma. Indolent lymphomas include MALT lymphoma, follicular lymphoma, duodenal-type follicular lymphoma, and small lymphocytic lymphoma. There was a statistically significant relationship between these two groups in terms of pathological subtypes and the endoscopic features (*p* < 0.05). In both groups, the predominant endoscopic manifestations were superficial type and protruding without ulcer type (69.4% in aggressive lymphomas and 81.6% in indolent lymphomas). Furthermore, fungating form and protruding with ulcer form were found in a higher proportion in aggressive lymphomas compared to indolent lymphomas (Table 6).

MALT lymphoma and DLBCL lesions were frequently manifested as the S-type, in 19/32 and 45/82 patients, respectively. S-type and P-type have been reported in almost all gastrointestinal lymphoma phenotypes. In addition to S-type, FL was also presented as PU type, SLL was P-type, and D-FL was P-type. The G-type was described in only three phenotypes, including MATL lymphoma, MCL, and DLBCL, while the MN-type suggested DLBCL or MALT lymphoma. There was a statistically significant correlation (*p* = 0.03) between the endoscopic features of GI lymphoma and the pathological subtypes. This difference was evident in the S-type group (*p* < 0.001). The *p*-value cannot be calculated for the other types owing to the low expected frequencies (Table 7).

**Figure 3:**
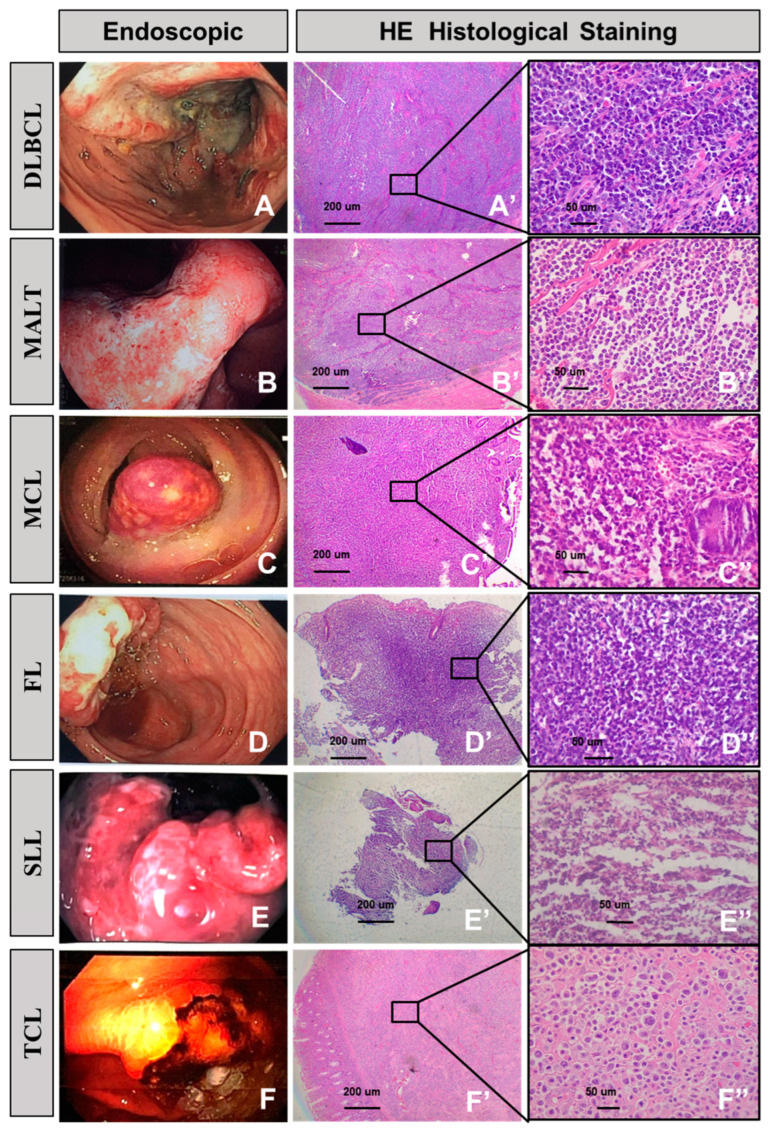
Endoscopic manifestation and corresponding histological features of gastrointestinal lymphoma. Diffuse large B-cell lymphoma (**A**,**A’**,**A”**). (**A**) Endoscopy revealing a fungating ulcer lesion. (**A’**) Transmural infiltration of large lymphoma cells. (**A”**) Most of the tumor cells resemble centroblasts and immunoblasts. Mucosa-associated lymphoid tissue (**B**,**B’**,**B”**). (**B**) Endoscopy reveals a superficial infiltration lesion. (**B’**) The diffuse lymphocytes infiltrate and extend into the submucosa. (**B”**) Monomorphic expansion of small lymphocytes. Mantle cell lymphoma (**C**,**C’**,**C”**). (**C**) Protruding without ulcerative lesions involving the colon. (**C’**) Low power, glandular structures are well-preserved. (**C”**) High power demonstrating a small- to medium-sized cell population. Follicular lymphoma (**D**,**D’**,**D”**). (**D**) Endoscopy revealed an ulcer in the ileocecum. (**D**’) Part follicular proliferation of centroblasts and centrocytes. (**D**”) Focal atypical follicle formation. Small lymphocyte lymphoma (**E**,**E’**,**E”**). (**E**) Endoscopy reveals protrusion with superficial ulcer involving rectum. (**E’**,**E”**) Neoplastic lymphocytes that are small, with scattered paraimmunoblasts. T cell lymphoma (**F**,**F’**,**F”**). (**F**) A gastric ulcer with hemorrhage on endoscopy. (**F’**,**F”**) The tumor cells show marked pleomorphism and a cytomorphological spectrum, ranging from medium to large to bizarre-appearing binucleated and multinucleated cells.

The associations between endoscopic findings and pathological features among our gastrointestinal lymphoma patient are demonstrated in Figure 3. In its legends, diffuse large B-cell lymphoma, mucosa-associated lymphoid tissue, mantle cell lymphoma, follicular lymphoma, small lymphocyte lymphoma, and T cell lymphoma have been described, for both endoscopic and histopathological results, respectively (Figure 3A–F).

## 4. Discussion

In our study, the stomach was the most frequent site of gastrointestinal lymphoma, followed by the colon, small intestine, ileocecum, rectum, and duodenum. This result was consistent with those of other studies indicating that the stomach is the main location of gastrointestinal lymphoma [7,9,15]. 

### 4.1. Helicobacter pylori Infection Is Associated with the Onset of Gastrointestinal Lymphoma

*H. pylori* was found in 38.7% of cases in our study. Patients with lymphomas of the stomach, ileocecum, or duodenum were more likely to suffer from *H. pylori* co-infection than those with lymphomas of other gastrointestinal organs. The link between *H. pylori* and gastric MALT lymphoma has been well documented, and *H. pylori* eradication has resulted in disease remission [16,17,18]. Our data also demonstrated a significant relationship between the infection of *H. pylori* and gastric MALT lymphoma (*p* < 0.05). Chronic *H. pylori* infection induces an antigenic stimulus, which results in clonal proliferation of B cells in the lymphoid tissue. This may lead to the development of MALT lymphoma [19,20,21]. 

Diffuse large B-cell lymphoma (DLBCL) is another type of gastric lymphoma associated with *H. pylori* infection. Both pure (de novo) DLBCL lymphoma type and MALT-transformed DLBCL-lymphoma (DLBCL-MALT) type have been shown to be related to *H. pylori* infection [22,23]. Numerous studies have shown that a significant percentage of patients with early-stage gastric de novo DLBCL and gastric DLBCL-MALT with *H. pylori* infection attain complete decline after receiving *H. pylori* eradication therapy [23,24,25]. 

On the other hand, the connection between *H. pylori* and lymphoma within the other parts of the gastrointestinal tract remains controversial. Numerous case reports have demonstrated a link between *H. pylori* infection and MALT lymphoma in the small and large intestines. Matsumoto and colleagues reported the case of a Japanese patient having colonic MALT lymphoma that regressed after *H. pylori* eradication [26]. In a systematic review study of Scott R. Kelley, there were eight cases of primary MALT lymphoma in the rectum that were solely treated with *H. pylori* eradication therapy. Results from this research showed a complete response that was eventually obtained in all cases after 3 weeks to 9 months [27]. Regarding the small intestine, Nagashima described a patient with MALT lymphoma in the duodenum that achieved complete regression of lymphoma after being treated with antibiotic drugs against *H. pylori* [28]. Another example in which the effect of elimination of *H. pylori* therapy on small bowel lymphoma reduction was demonstrated in a patient with MALT lymphoma in the stomach and duodenum that was synchronous with multiple gastric carcinomas [29]. These results provide assistance for the causal role of *H. pylori* in extragastric MALT lymphoma of the gastrointestinal tract. 

### 4.2. Endoscopic Findings of Gastrointestinal Lymphoma

The standard endoscopic categorization of gastrointestinal lymphoma has not yet been defined. Previous studies have proposed different classifications to analyze the endoscopic features of the disease (Table 8). In this work, we used an endoscopic categorization based on the proposal of T. Kanno and colleagues which is easier to carry out compared to other classifications. This category includes six patterns: superficial form, protruding without ulcer form, fungating form, protruding with ulcer form, giant fold form, and multiple nodule form [3]. 

Herein, we separated different types of gastrointestinal lymphoma into two groups, indolent lymphoma and aggressive lymphoma, to recognize the endoscopic manifestations related to each group. The indolent lymphoma group included MALT lymphoma, follicular lymphoma, duodenal-type follicular lymphoma, and small lymphocytic lymphoma. The aggressive form group contained diffuse large B cell lymphoma, mantle cell lymphoma (mantle cell lymphoma is considered as an aggressive subtype of B-cell non-Hodgkin lymphoma with generally poor prognosis [33]), and T-cell lymphoma. In both groups, the predominant endoscopic manifestations were superficial type and protruding without ulcer type. We found that fungating type and protruding with ulcer type were highly suggestive of aggressive lymphomas. This was statistically significant (*p* < 0.05). 

### 4.3. Endoscopic Features of Diffuse Large B Cell Lymphoma (DLBCL)

Diffuse large B cell lymphoma of the GI tract is an aggressive lymphoma that may originate de novo or through the transformation of another lymphoma, most frequently MALT lymphoma [2]. DLBCL is the most prevalent pathological form of digestive tract lymphoma. In our investigation, this lymphoma was predominantly found in the stomach (59/83 cases), followed by the large intestine (19/83 cases) and small intestine (5/83 cases). 

Various endoscopic manifestations of gastric DLBCL have been reported. Most DLBCL lymphomas in our study were superficial. In another study, the tumor manifested as polypoid, nodular, ulcerative, erosive, diffusely infiltrating, thickened fold-like, and mixed types [34,35]. Kyoungwon Jung discovered no statistically significant differences between primary and secondary gastric diffuse large B cell lymphoma under endoscopy. The ulcero-infiltrative type predominated in both groups, followed by the mixed form [36]. 

In our study, colorectal DLBCL lymphomas manifested as a protruding type with or without ulcer (which was consistent with the polypoid type). This was similar to a study by Yachida that reported that in 48 cases of DLBCL lymphoma in the large intestine, and 52% of lesions showed a polypoid appearance via endoscopy, and 38% were of the ulcerative type [37]. Yoshifumi Hori also found that the polypoid and ulcerative types were predominant in colorectal DLBCL lymphomas, accounting for 48% and 44%, respectively [30]. DLBCL in the small intestine is rare with only a few reports. Endoscopic findings are predominant for polypoid type and ulcerative type [38,39]. 

### 4.4. Endoscopic Features of MALT Lymphoma

MALT lymphoma is a low-grade non-Hodgkin lymphoma [40]. Approximately 70% of MALT lymphoma cases occur in the stomach, followed by the small intestine, colon, and rectum [2]. In this study, MALT lymphoma was predominantly observed in the stomach (22/32 patients), followed by the small intestine (7/32 patients), and large intestine (3/32 patients). The endoscopic appearance of MALT lymphoma in the stomach was of the superficial type, while in the small intestine and large intestine, it was of the superficial and protruding types.

The endoscopic characteristics of gastric MALT lymphoma are not specific and can mimic benign diseases such as erosions, gastritis, or gastric adenocarcinoma [41]. In previous studies, endoscopic findings in this disease were mainly superficial, accounting for nearly 70–80% of cases, in addition to other types such as ulcerative and mass-forming [31,42]. Nevertheless, in other investigations, the most frequent macroscopic finding of gastric MALT lymphoma was ulcerative [5,43,44]. 

Regarding MALT lymphoma of the small intestine, ulcerative type, or polypoid lesions are often identified by endoscopic examination [28,45]. Colorectal MALT lymphoma can have different macroscopic manifestations, including elevated, polypoid lesions with intact mucosal aspect or erosion at endoscopy [46,47]. 

Furthermore, the enhanced endoscopy technologies, including narrow-band imaging and zoom function to magnify the micro pattern of surface and vessels, together with chemical dyes like indigo carmine can provide endoscopists clearer and detailed pictures of typical lymphoma, such as tree-like or branch-like microvessel pattern. These techniques may enable us to detect and characterize the lesions, orient the biopsies, and possibly contribute significantly to the final diagnosis (Figure 2).

### 4.5. Endoscopic Features of Mantle Cell Lymphomas (MCL)

Primary mantle cell lymphoma (MCL) of the gastrointestinal tract is uncommon, and its standard treatment is unclear. In our study, mantle cell lymphoma was located mostly in the large intestine (9/15 cases), most of which presented as the protruding type via endoscopy. According to previous studies, the digestive tract has been identified as one of the most common extranodal locations for MCL, with the colon being the organ that is usually affected by the condition [48,49]. Macroscopically, large masses, superficial ulcers, and diffuse thickening of the mucosa may be observed; however, polyp type is the most frequent endoscopic manifestation of MCL in the gastrointestinal tract, and some MCL patients also have polyposis lesions [48,50,51]. The term “multiple lymphomatous polyposis” was coined by Cornes in 1961 to characterize the presence of numerous polypoid lesions along the gastrointestinal tract that are caused by malignant lymphoma involving the mucosa [52]. Endoscopy of the upper gastrointestinal tract and colonoscopy are important diagnostic instruments for multiple lymphomatous polyposis Macroscopically to identify polyps location and obtain tissue biopsy specimens. The endoscopic or radiological evaluation alone cannot differentiate lymphomatous polyposis from adenomatous or hamartomatous polyposis, and therefore, tissue diagnosis is necessary. In addition, not all cases of lymphomatous polyposis of the digestive tract are caused by MCL. Multiple lymphomatous polyposis has also been observed in MALT and follicular lymphomas [37,53].

### 4.6. Endoscopic Features of Rare Types of Lymphoma 

In our study, T-cell lymphoma (four cases), follicular lymphoma (two cases), duodenal-type follicular lymphoma (two cases), and small lymphocytic lymphoma (two cases) accounted for a small portion. 

These T-cell lymphoma were found in both the stomach and intestine (one case in the small intestine, one case in the stomach, and two cases in the large intestine). The lesions manifested as fungating form, superficial form, protruding without ulcer form, and protruding with ulcer form in one case each. According to Sugita et al., the duodenum and jejunum are the organs most affected by T cell lymphoma in the gastrointestinal tract, followed by the ileum and colon. However, the stomach is less relevant [54]. Previous research has demonstrated that T-cell lymphomas frequently exhibit ulcerative or ulceroinfiltrating lesions [55,56,57].

Gastrointestinal follicular lymphoma is an uncommon entity, accounting for less than 4% of gastrointestinal non-Hodgkin lymphoma [58]. It is a low-grade lymphoma that typically progresses slowly. In our study, only two cases of follicular lymphoma were found (one in the large intestine and one in the stomach), in addition to two cases of duodenal-type follicular lymphoma. Endoscopy has become more prevalent in hospitals and clinics, leading to an increase in the number of published studies of gastrointestinal follicular lymphoma. In a study by Takata et al. in 125 patients, follicular lymphoma was most frequently located in the duodenum, followed by the ileum, jejunum, rectum, and colon. The stomach was the last location of this lymphoma [59]. The same distribution was also found in other studies [55,60,61]. Nodular lesion was the most frequent endoscopic finding among patients with gastrointestinal follicular lymphoma (71–80%), and tumors involving the second part of the duodenum exhibited this macroscopic appearance [55,59]. Tumors of the stomach or colon exhibited a range of morphologies, including tumor-like, flat-elevated, and ulcerative [32,62]. Since the 2016 WHO classification, duodenal-type follicular lymphoma has been recognized as a variant of follicular lymphoma [8]. This is a B lymphocyte tumor with follicular architecture typically seen in the second part of the duodenum, with low-grade progression as well as good prognosis.

The digestive tract is infrequently affected by small lymphocytic lymphoma/chronic lymphocytic leukemia/(SLL/CLL), which are characterized by the diffuse infiltration of small lymphocytes devoid of follicle formation or lymphoepithelial lesions [63]. Some cases of primary SLL have been reported, mostly in the colon. The endoscopic findings in these patients were multiple small nodules or polyp type lesions [63,64].

## 5. Conclusions

In conclusion, our study provides more information on the endoscopic and pathological characteristics of different types of gastrointestinal lymphomas. This study showed that most subtypes of gastrointestinal non-Hodgkin lymphomas exhibited same endoscopic features (superficial type and ulcer type). Aggressive gastrointestinal non-Hodgkin lymphomas (diffuse large B-cell lymphoma, mantle cell lymphoma, and T-cell lymphoma) were highly suspected when fungating lesions and protruding with ulcer lesions were encountered under endoscopy. Therefore, endoscopists must be aware of the associations between the enhanced endoscopic characteristics and histopathological features of gastrointestinal lymphoma to recognize and characterize suspected lesions as well as to perform appropriate biopsies.

## Figures and Tables

**Table 1:** Lesion location distribution and tumor size of gastrointestinal lymphoma.

Lesion Location	Case Number		Tumor Size (mm)	
	*n*	%	Mean	SD
Stomach	86	61.4	37.9	18.3
Colon	14	10.0	45.8	37.2
Small intestine	14	10.0	43.4	27.9
Ileocecum	12	8.6	40.2	17.8
Rectum	9	6.4	46.3	26.7
Duodenum	5	3.6	24.0	6.5
Total	140	100.0	39.5	22.2

**Table 2:** The endoscopic features of gastrointestinal lymphoma in Vietnam.

Endoscopic Features	Case Number	%
S	72	51.4
P	29	20.7
F	18	12.9
PU	13	9.3
G	5	3.6
MN	3	2.1
Total	140	100.0

Note: S: Superficial form; P: Protruding without ulcer form; F: Fungating form; PU: Protruding with Ulcer form; G: Giant fold form; MN: Multiple Nodule form.

**Table 3:** Relationship between lesion location and *H. pylori* infection.

Lesion Location	*H. pylori* Negative		*H. pylori* Positive		*p*
	*n*	%	*n*	%	
Stomach	41	49.4	42	50.6	1
Colon	12	100.0	0	0.0	**0.001**
Small intestine	10	90.9	1	9.1	**0.016**
Ileocecum	5	62.5	3	37.5	0.727
Rectum	5	100.0	0	0.0	0.063
Duodenum	3	60.0	2	40.0	-
Total	76	61.3	48	38.7	**0.015**

Bold: the *p*-value is statistically significant.

**Table 4:** Relationship between the pathological subtype of gastrointestinal lymphoma and *H. pylori* infection.

GI Lymphoma Subtypes		*H. pylori* Negative		*H. pylori* Positive		*p*
		*n*	%	*n*	%	
DLBCL		48	64.0	27	36.0	**0.020**
MALT lymphoma	Total	15	46.9	17	53.1	0.860
	Stomach	7	31.8	15	68.2	**0.021**
	Other locations	8	80.0	2	20.0	
MCL		8	72.7	3	27.3	0.388
FL		0	0.0	1	100.0	1
SLL		1	100.0	0	0.0	1
D-FL		2	100.0	0	0.0	0.5
TCL		2	100.0	0	0.0	0.5
Total		76	61.3	48	38.7	0.103

Note: DLBCL—diffuse large B-cell lymphoma; MALT—mucosa-associated lymphoid tissue; MCL—mantle cell lymphoma; FL—follicular lymphoma; SLL—small lymphocytic lymphoma; D-FL—duodenal-type follicular lymphoma; TCL—T-cell lymphoma. Bold: the *p*-value is statistically significant.

**Table 5:** Relationship between lesion location and pathological subtypes.

Lesion Location	Pathological Subtypes *n* (%)							*p*
	DLBCL	MALT Lymphoma	MCL	FL	SLL	D-FL	TCL	
Stomach	59 (68.7)	22 (25.6)	2 (2.4)	1 (1.2)	1 (1.2)	0 (0.0)	1 (1.2)	**<0.001**
Colon	9 (64.3)	2 (14.3)	2 (14.3)	0 (0.0)	0 (0.0)	0 (0.0)	1 (7.2)	-
Small intestine	5 (28.6)	5 (35.8)	3 (21.5)	0 (0.0)	0 (0.0)	0 (0.0)	1 (7.2)	-
Ileocecum	6 (50.0)	0 (0.0)	4 (33.4)	1 (8.4)	0 (0.0)	0 (0.0)	1 (8.4)	-
Rectum	4 (44.5)	1 (11.2)	3 (33.4)	0 (0.0)	1 (11.2)	0 (0.0)	0 (0.0)	-
Duodenum	0 (0.0)	2 (40)	1 (20.0)	0 (0.0)	0 (0.0)	2 (40)	0 (0.0)	-
Total	83 (58.6)	32 (22.9)	15 (10.8)	2 (1.5)	2 (1.5)	2 (1.5)	4 (2.9)	**<0.001**

Note: DLBCL—diffuse large B-cell lymphoma; MALT—mucosa-associated lymphoid tissue; MCL—mantle cell lymphoma; FL—follicular lymphoma; SLL—small lymphocytic lymphoma; D-FL—duodenal-type follicular lymphoma; TCL—T-cell lymphoma. Bold: the *p*-value is statistically significant.

**Table 6:** Association between endoscopic features and pathological subtypes (aggressive types and indolent types).

Endoscopic Features	Pathological Subtypes *n* (%)		*p*
	Aggressive Lymphoma	Indolent Lymphoma	
S	49 (50.0)	22 (57.9)	**0.018**
P	19 (19.4)	9 (23.7)	
F	16 (16.3)	1 (2.6)	
PU	11 (11.2)	1 (2.6)	
G	2 (2.0)	3 (7.9)	
MN	1 (1.0)	2 (5.3)	
Total	102	38	

Note: S: Superficial form; P: Protruding without ulcer form; F: Fungating form; PU: Protruding with Ulcer form; G: Giant fold form; MN: Multiple Nodule form. Bold: the *p*-value is statistically significant.

**Table 7:** Association between endoscopic features and pathological subtypes.

Endoscopic Features	Pathological Subtypes *n* (%)							*p*
	DLBCL	MALT Lymphoma	MCL	FL	SLL	D-FL	TCL	
S	45 (55.6)	19 (59.6)	4 (26.8)	1 (50)	1 (50.0)	1 (50)	1 (25)	**<0.001**
P	13 (15.6)	7 (21.8)	6 (39.7)	0 (0.0)	1 (50.0)	1 (50)	1 (25)	-
F	15 (18.0)	1 (3.1)	1 (6.7)	0 (0.0)	0 (0.0)	0 (0.0)	1 (25)	-
PU	9 (8.4)	0 (0.0)	3 (20.1)	1 (50)	0 (0.0)	0 (0.0)	1 (25)	-
G	1 (1.2)	3 (9.3)	1 (6.7)	0 (0.0)	0 (0.0)	0 (0.0)	0 (0.0)	-
MN	1 (1.2)	2 (6.2)	0 (0.0)	0 (0.0)	0 (0.0)	0 (0.0)	0 (0.0)	-
Total	83	32	15	2	2	2	4	**0.03**

Note: S: Superficial form; P: Protruding without ulcer form; F: Fungating form; PU: Protruding with Ulcer form; G: Giant fold form; MN: Multiple Nodule form. Bold: the *p*-value is statistically significant.

**Table 8:** Endoscopic classifications of gastrointestinal lymphoma.

Authors	Endoscopic Classification	Histological Types of GI Lymphoma	Sites
T. Kanno (*n* = 63) [3]	1. Superficial type. 2. Protruding without ulceration type. 3. Protruding with ulceration type. 4. Fungating type. 5. Multiple nodules type. 6. Giant fold type	GI lymphomas not including lymphoma (MALT)	Stomach, small intestine, and large intestine
Yoshifumi Hori (*n* = 25) [30]	Polypoid lesion, ulcerative lesion, lymphomatous polyposis lesion, diffuse-infiltrating lesion, and mixed lesion	Diffuse large B-cell lymphoma	Colorectum
Angelo Zullo (*n* = 2000) [5]	Exophytic type (polypoid mass); ulcerative type (single or multiple ulcerations); hypertrophic type (large or giant folds, nodular pattern), mixed type	MALT lymphoma, diffuse large B-cell lymphoma	Stomach
Eun Jeong Gong (*n* = 345) [31]	Superficial, ulcerative or mass-forming type	MALT lymphoma	Stomach
Katsuyoshi Takata (*n* = 125) [32]	Multiple nodules, superficial, polypoid, ulcerative, diffuse, and unclassified	Follicular lymphoma	Stomach, small intestine, and large intestine
Nakamura (*n* = 197) [6]	superficial-spreading, followed by mass-forming, diffusely infiltrating and other types	B-cell lymphomas	Stomach
This study	1. Superficial type. 2. Protruding without ulceration type. 3. Protruding with ulceration type. 4. Fungating type. 5. Multiple nodules type. 6. Giant fold type	DLBCL—diffuse large B-cell lymphoma; MALT—mucosa-associated lymphoid tissue; MCL—mantle cell lymphoma; FL—follicular lymphoma; SLL—small lymphocytic lymphoma; D-FL—duodenal-type follicular lymphoma; TCL—T-cell lymphoma	Stomach, small intestine, and large intestine

## Data Availability

This article is belong to Research Projects in Science and Technology of the Vietnamese Ministry of Education and Training that has not been finished. Therefore, due to the security policy, we are not able to share the research data.

## References

[1] Ghimire P., Wu G.Y., Zhu L. (2011). Primary gastrointestinal lymphoma. World J. Gastroenterol..

[2] Bautista-Quach M.A., Ake C.D., Chen M., Wang J. (2012). Gastrointestinal lymphomas: Morphology, immunophenotype and molecular features. J. Gastrointest. Oncol..

[3] Kanno T., Katano T., Shimura T., Nishigaki R., Kojima Y., Sasaki M., Okuda Y., Sugimura N., Fukusada S., Mizuno Y. (2022). Characteristic endoscopic findings of gastrointestinal malignant lymphomas other than mucosa-associated lymphoid tissue lymphoma. Acta Gastro-Enterol. Belg..

[4] Hu Q., Zhang Y., Zhang X., Fu K. (2016). Gastric mucosa-associated lymphoid tissue lymphoma and Helicobacter pylori infection: A review of current diagnosis and management. Biomark. Res..

[5] Zullo A., Hassan C., Andriani A., Cristofari F., Cardinale V., Spinelli G.P., Tomao S., Morini S. (2010). Primary low-grade and high-grade gastric MALT-lymphoma presentation. J. Clin. Gastroenterol..

[6] Nakamura S., Matsumoto T. (2013). Gastrointestinal lymphoma: Recent advances in diagnosis and treatment. Digestion.

[7] Xiang Y., Yao L. (2022). Analysis of 78 Cases of Primary Gastrointestinal Lymphoma. J. Healthc. Eng..

[8] Swerdlow S.H., Campo E., Pileri S.A., Harris N.L., Stein H., Siebert R., Advani R., Ghielmini M., Salles G.A., Zelenetz A.D. (2016). The 2016 revision of the World Health Organization classification of lymphoid neoplasms. Blood.

[9] Erkut M., Erkut N., Bektaş Ö., Fidan S., Coşar A.M., Sönmez M. (2022). Effect of Clinical, Endoscopic, Radiological Findings, and Complications on Survival in Patients with Primary Gastrointestinal Lymphoma. Turk. J. Gastroenterol. Off. J. Turk. Soc. Gastroenterol..

[10] Juárez-Salcedo L.M., Sokol L., Chavez J.C., Dalia S. (2018). Primary Gastric Lymphoma, Epidemiology, Clinical Diagnosis, and Treatment. Cancer Control J. Moffitt Cancer Cent..

[11] Alvarez-Lesmes J., Chapman J., Cassidy D., Zhou Y., Garcia-Buitrago M., Montgomery E., Lossos I., Sussman D., Poveda J. (2021). Gastrointestinal Tract Lymphomas: A Review of the Most Commonly Encountered Lymphomas. Arch. Pathol. Lab. Med..

[12] Dawson I.M., Cornes J.S., Morson B.C. (1961). Primary malignant lymphoid tumours of the intestinal tract. Report of 37 cases with a study of factors influencing prognosis. Br. J. Surg..

[13] WHO Classification of Tumours (2019). Digestive System Tumours: WHO Classification of Tumours, Volume 1.

[14] Posit Team (2022). R Studio: Integrated Development Environment for R. http://www.posit.co/.

[15] Chen L., Kan Y., Wang X., Ge P., Ding T., Zhai Q., Wang Y., Yu Y., Wang X., Zhao Z. (2020). Overexpression of microRNA-130a predicts adverse prognosis of primary gastrointestinal diffuse large B-cell lymphoma. Oncol. Lett..

[16] Grgov S., Katić V., Krstić M., Nagorni A., Radovanović-Dinić B., Tasić T. (2015). Treatment of low-grade gastric MALT lymphoma using Helicobacter pylori eradication. Vojnosanit. Pregl..

[17] Sung-Hsin K., Kun-Huei Y., Chung-Wu L., Li-Tzong C., Ming-Shiang W., Ann-Lii C., Bruna Maria R. (2021). Revisiting the Full Spectrum of *Helicobacter pylori*-Related Gastric Lymphoma. Helicobacter Pylori.

[18] Keikha M., Sahebkar A., Yamaoka Y., Karbalaei M. (2022). Helicobacter pylori cagA status and gastric mucosa-associated lymphoid tissue lymphoma: A systematic review and meta-analysis. J. Health Popul. Nutr..

[19] Umehara S., Higashi H., Ohnishi N., Asaka M., Hatakeyama M. (2003). Effects of Helicobacter pylori CagA protein on the growth and survival of B lymphocytes, the origin of MALT lymphoma. Oncogene.

[20] Shaye O.S., Levine A.M. (2006). Marginal zone lymphoma. J. Natl. Compr. Cancer Netw. JNCCN.

[21] Krisch L.M., Posselt G., Hammerl P., Wessler S. (2016). CagA Phosphorylation in Helicobacter pylori-Infected B Cells Is Mediated by the Nonreceptor Tyrosine Kinases of the Src and Abl Families. Infect. Immun..

[22] Paydas S. (2015). Helicobacter pylori eradication in gastric diffuse large B cell lymphoma. World J. Gastroenterol..

[23] Cavanna L., Pagani R., Seghini P., Zangrandi A., Paties C. (2008). High grade B-cell gastric lymphoma with complete pathologic remission after eradication of Helicobacter pylori infection: Report of a case and review of the literature. World J. Surg. Oncol..

[24] Nakamura S., Matsumoto T., Suekane H., Takeshita M., Hizawa K., Kawasaki M., Yao T., Tsuneyoshi M., Iida M., Fujishima M. (2001). Predictive value of endoscopic ultrasonography for regression of gastric low grade and high grade MALT lymphomas after eradication of Helicobacter pylori. Gut.

[25] Ferreri A.J., Govi S., Raderer M., Mulè A., Andriani A., Caracciolo D., Devizzi L., Ilariucci F., Luminari S., Viale E. (2012). Helicobacter pylori eradication as exclusive treatment for limited-stage gastric diffuse large B-cell lymphoma: Results of a multicenter phase 2 trial. Blood.

[26] Matsumoto T., Iida M., Shimizu M. (1997). Regression of mucosa-associated lymphoid-tissue lymphoma of rectum after eradication of Helicobacter pylori. Lancet.

[27] Kelley S.R. (2017). Mucosa-associated lymphoid tissue (MALT) variant of primary rectal lymphoma: A review of the English literature. Int. J. Color. Dis..

[28] Nagashima R., Takeda H., Maeda K., Ohno S., Takahashi T. (1996). Regression of duodenal mucosa-associated lymphoid tissue lymphoma after eradication of Helicobacter pylori. Gastroenterology.

[29] Yokoyama T., Tanaka T., Harada S., Ueda T., Ejiri G., Sasaki S., Takeda M., Yoshimura A. (2021). A case of gastric and duodenal mucosa-associated lymphoid tissue lymphoma with multiple gastric cancers: A case report. Surg. Case Rep..

[30] Hori Y., Yamamoto H., Nozaki Y., Torisu T., Fujiwara M., Taguchi K., Nishiyama K., Nakamura S., Kitazono T., Oda Y. (2020). Colorectal diffuse large B-cell lymphoma: Molecular subclassification and prognostic significance of immunoglobulin gene translocation. Hum. Pathol..

[31] Gong E.J., Ahn J.Y., Jung H.Y., Park H., Ko Y.B., Na H.K., Jung K.W., Kim D.H., Lee J.H., Choi K.D. (2016). Helicobacter pylori Eradication Therapy Is Effective as the Initial Treatment for Patients with H. pylori-Negative and Disseminated Gastric Mucosa-Associated Lymphoid Tissue Lymphoma. Gut Liver.

[32] Takata K., Okada H., Ohmiya N., Nakamura S., Kitadai Y., Tari A., Akamatsu T., Kawai H., Tanaka S., Araki H. (2011). Primary gastrointestinal follicular lymphoma involving the duodenal second portion is a distinct entity: A multicenter, retrospective analysis in Japan. Cancer Sci..

[33] Skarbnik A.P., Goy A.H. (2015). Mantle cell lymphoma: State of the art. Clin. Adv. Hematol. Oncol. HO.

[34] Zepeda-Gomez S. (2008). Gastric infiltration of diffuse large B-cell lymphoma: Endoscopic diagnosis and improvement of lesions after chemotherapy. World J. Gastroenterol..

[35] Vetro C., Romano A., Amico I., Conticello C., Motta G., Figuera A., Chiarenza A., Di Raimondo C., Giulietti G., Bonanno G. (2014). Endoscopic features of gastro-intestinal lymphomas: From diagnosis to follow-up. World J. Gastroenterol..

[36] Jung K., Jeon H.S., Park M.I., Choe I.H., Je H.S., Kim J.H., Kim S.E., Moon W., Park S.J. (2020). Differences in Endoscopic Findings of Primary and Secondary Gastric Lymphoma. KMJ.

[37] Yachida T., Matsuda T., Sakamoto T., Nakajima T., Kakugawa Y., Maeshima A.M., Taniguchi H., Kushima R., Tobinai K., Kobara H. (2022). Endoscopic features of colorectal lymphoma according to histological type. JGH Open Open Access J. Gastroenterol. Hepatol..

[38] Nakamura S., Matsumoto T., Takeshita M., Kurahara K., Yao T., Tsuneyoshi M., Iida M., Fujishima M. (2000). A clinicopathologic study of primary small intestine lymphoma: Prognostic significance of mucosa-associated lymphoid tissue-derived lymphoma. Cancer.

[39] Catalano C., Sidhu L., Anyadike N., Syed U.M., Companioni R.A.C., Tiba M., Tomaino C. (2015). Diffuse Large B-Cell Lymphoma (DLBCL) of the Small Bowel Presenting as Acute Gastroenteritis: 1096. Off. J. Am. Coll. Gastroenterol.|ACG.

[40] Press O.W., Lichtman M.A., Press O.W., Lichtman M.A., Leonard J.P. (2017). General Considerations oF Lymphomas: Epidemiology, Etiology, Heterogeneity, and Primary Extranodal Disease. Williams Hematology Malignant Lymphoid Diseases.

[41] Inagaki H., Nakamura T., Li C., Sugiyama T., Asaka M., Kodaira J., Iwano M., Chiba T., Okazaki K., Kato A. (2004). Gastric MALT lymphomas are divided into three groups based on responsiveness to Helicobacter Pylori eradication and detection of API2-MALT1 fusion. Am. J. Surg. Pathol..

[42] Nakamura S., Sugiyama T., Matsumoto T., Iijima K., Ono S., Tajika M., Tari A., Kitadai Y., Matsumoto H., Nagaya T. (2012). Long-term clinical outcome of gastric MALT lymphoma after eradication of Helicobacter pylori: A multicentre cohort follow-up study of 420 patients in Japan. Gut.

[43] Andriani A., Zullo A., Di Raimondo F., Patti C., Tedeschi L., Recine U., Caruso L., Bonanno G., Chiarenza A., Lizzani G. (2006). Clinical and endoscopic presentation of primary gastric lymphoma: A multicentre study. Aliment. Pharmacol. Ther..

[44] Cui X., Zhou T., Jiang D., Liu H., Wang J., Yuan S., Li H., Yan P., Gao Y. (2017). Clinical manifestations and endoscopic presentations of gastric lymphoma: A multicenter seven year retrospective survey. Rev. Esp. Enfermedades Dig..

[45] Terada T. (2013). Extranodal marginal zone lymphoma of mucosa-associated lymphoid tissue (MALT lymphoma) of the ileum in a 35-year-old Japanese woman. Int. J. Clin. Exp. Pathol..

[46] Ahlawat S., Kanber Y., Charabaty-Pishvaian A., Ozdemirli M., Cohen P., Benjamin S., Haddad N. (2006). Primary mucosa-associated lymphoid tissue (MALT) lymphoma occurring in the rectum: A case report and review of the literature. South. Med. J..

[47] Akasaka R., Chiba T., Dutta A.K., Toya Y., Mizutani T., Shozushima T., Abe K., Kamei M., Kasugai S., Shibata S. (2012). Colonic mucosa-associated lymphoid tissue lymphoma. Case Rep. Gastroenterol..

[48] Vetro C., Bonanno G., Giulietti G., Romano A., Conticello C., Chiarenza A., Spina P., Coppolino F., Cunsolo R., Raimondo F.D. (2015). Rare gastrointestinal lymphomas: The endoscopic investigation. World J. Gastrointest. Endosc..

[49] Lee H.H., Cho S.G., Lee I.S., Cho H.J., Jeon Y.W., O J.H., Jung S.E., Choi B.O., Park K.S., Yang S.W. (2020). Mantle cell lymphoma with gastrointestinal involvement and the role of endoscopic examinations. PLoS ONE.

[50] Romaguera J.E., Medeiros L.J., Hagemeister F.B., Fayad L.E., Rodriguez M.A., Pro B., Younes A., McLaughlin P., Goy A., Sarris A.H. (2003). Frequency of gastrointestinal involvement and its clinical significance in mantle cell lymphoma. Cancer.

[51] Castellino A., Tun A.M., Wang Y., Habermann T.M., King R.L., Ristow K.M., Cerhan J.R., Inwards D.J., Paludo J., Ansell S.M. (2021). Clinical characteristics and outcomes of primary versus secondary gastrointestinal mantle cell lymphoma. Blood Cancer J..

[52] Cornes J.S. (1961). Multiple lymphomatous polyposis of the gastrointestinal tract. Cancer.

[53] Aiman S., Chakrapani A., Sawaimoon S., Sen S., Chandy M., Chatterjee S. (2015). Multiple lymphomatous polyposis: Characteristic endoscopic features. Indian J. Gastroenterol..

[54] Sugita S., Iijima T., Furuya S., Kano J., Yanaka A., Ohta K., Kojima H., Noguchi M. (2007). Gastric T-cell lymphoma with cytotoxic phenotype. Pathol. Int..

[55] Shia J., Teruya-Feldstein J., Pan D., Hegde A., Klimstra D.S., Chaganti R.S., Qin J., Portlock C.S., Filippa D.A. (2002). Primary follicular lymphoma of the gastrointestinal tract: A clinical and pathologic study of 26 cases. Am. J. Surg. Pathol..

[56] Kim Y.H., Lee J.H., Yang S.K., Kim T.I., Kim J.S., Kim H.J., Kim J.I., Kim S.W., Kim J.O., Jung I.K. (2005). Primary colon lymphoma in Korea: A KASID (Korean Association for the Study of Intestinal Diseases) Study. Dig. Dis. Sci..

[57] Kim D.H., Lee D., Kim J.W., Huh J., Park S.H., Ha H.K., Suh C., Yoon S.M., Kim K.J., Choi K.D. (2014). Endoscopic and clinical analysis of primary T-cell lymphoma of the gastrointestinal tract according to pathological subtype. J. Gastroenterol. Hepatol..

[58] Yamamoto S., Nakase H., Yamashita K., Matsuura M., Takada M., Kawanami C., Chiba T. (2010). Gastrointestinal follicular lymphoma: Review of the literature. J. Gastroenterol..

[59] Takata K., Miyata-Takata T., Sato Y., Iwamuro M., Okada H., Tari A., Yoshino T. (2018). Gastrointestinal follicular lymphoma: Current knowledge and future challenges. Pathol. Int..

[60] Damaj G., Verkarre V., Delmer A., Solal-Celigny P., Yakoub-Agha I., Cellier C., Maurschhauser F., Bouabdallah R., Leblond V., Lefrère F. (2003). Primary follicular lymphoma of the gastrointestinal tract: A study of 25 cases and a literature review. Ann. Oncol. Off. J. Eur. Soc. Med. Oncol..

[61] Misdraji J., Harris N.L., Hasserjian R.P., Lauwers G.Y., Ferry J.A. (2011). Primary follicular lymphoma of the gastrointestinal tract. Am. J. Surg. Pathol..

[62] Kodama M., Kitadai Y., Shishido T., Shimamoto M., Fukumoto A., Masuda H., Tanaka S., Yoshihara M., Sakai A., Nakayama H. (2008). Primary follicular lymphoma of the gastrointestinal tract: A retrospective case series. Endoscopy.

[63] Aje K.T., Abegunde A.T., Mirza K. (2022). Intestinal Infiltration of Chronic Lymphocytic Leukemia/Small Lymphocytic Lymphoma Found on Screening Colonoscopy. Cureus.

[64] Dambowy P.R., Chaudhary N.A. (2006). Chronic Lymphocytic Leukemia (Small Lymphocytic Lymphoma) Involving the Colon: 919. Off. J. Am. Coll. Gastroenterol.|ACG.

